# Antioxidant Effect of a Probiotic Product on a Model of Oxidative Stress Induced by High-Intensity and Duration Physical Exercise

**DOI:** 10.3390/antiox10020323

**Published:** 2021-02-22

**Authors:** Maravillas Sánchez Macarro, Vicente Ávila-Gandía, Silvia Pérez-Piñero, Fernando Cánovas, Ana María García-Muñoz, María Salud Abellán-Ruiz, Desirée Victoria-Montesinos, Antonio J. Luque-Rubia, Eric Climent, Salvador Genovés, Daniel Ramon, Empar Chenoll, Francisco Javier López-Román

**Affiliations:** 1 Department of Exercise Physiology, San Antonio Catholic University of Murcia (UCAM), 30107 Murcia, Spain; msanchez4@ucam.edu (M.S.M.); vavila@ucam.edu (V.Á.-G.); sperez2@ucam.edu (S.P.-P.); fcanovas@ucam.edu (F.C.); amgarcia13@ucam.edu (A.M.G.-M.); msabellan@ucam.edu (M.S.A.-R.); dvictoria@ucam.edu (D.V.-M.); ajluque@ucam.edu (A.J.L.-R.); 2 Research and Development Department, ADM-Biopolis, ADM, Parc Cientific Universitat de Valencia, Paterna, 46980 Valencia, Spain; Eric.Climent@adm.com (E.C.); salvador.genoves@adm.com (S.G.); Daniel.RamonVidal@adm.com (D.R.); Maria.Chenoll@adm.com (E.C.); 3 Primary Care Research Group, Biomedical Research Institute of Murcia (IMIB-Arrixaca), 30120 Murcia, Spain

**Keywords:** oxidative stress, probiotics, physical exercise, male cyclists, oxidative stress biomarkers, antioxidative enzymes

## Abstract

This randomized double-blind and controlled single-center clinical trial was designed to evaluate the effect of a 6-week intake of a probiotic product (1 capsule/day) vs. a placebo on an oxidative stress model of physical exercise (high intensity and duration) in male cyclists (probiotic group, *n* = 22; placebo, *n* = 21). This probiotic included three lyophilized strains (*Bifidobacterium longum* CECT 7347, *Lactobacillus casei* CECT 9104, and *Lactobacillus rhamnosus* CECT 8361). Study variables were urinary isoprostane, serum malondialdehyde (MDA), serum oxidized low-density lipoprotein (Ox-LDL), urinary 8-hydroxy-2′-deoxiguanosine (8-OHdG), serum protein carbonyl, serum glutathione peroxidase (GPx), and serum superoxide dismutase (SOD). At 6 weeks, as compared with baseline, significant differences in 8-OHdG (Δ mean difference −10.9 (95% CI −14.5 to −7.3); *p* < 0.001), MDA (Δ mean difference −207.6 (95% CI −349.1 to −66.1; *p* < 0.05), and Ox-LDL (Δ mean difference −122.5 (95% CI −240 to −4.5); *p* < 0.05) were found in the probiotic group only. Serum GPx did not increase in the probiotic group, whereas the mean difference was significant in the placebo group (477.8 (95% CI 112.5 to 843.2); *p* < 0.05). These findings suggest an antioxidant effect of this probiotic on underlying interacting oxidative stress mechanisms and their modulation in healthy subjects. The study was registered in ClinicalTrials.gov (NCT03798821).

## 1. Introduction

Oxidative stress is characterized by the inability of the organism to detoxify reactive oxygen species (ROS) caused by a disequilibrium in the balance between their production and accumulation in cells and tissues. ROS generated by biological systems as metabolic by-products include hydrogen peroxide, superoxide and hydroxyl radicals, and singlet oxygen [1]. The oxidation products or nitrosylated products linked to ROS have a variety of detrimental effects on crucial cellular functions. Cell enzymatic antioxidant defensive systems include superoxide dismutase (SOD), catalase (CAT), glutathione reductase, and glutathione peroxidase (GPx) as the most important scavengers [2,3]. On the other hand, overproduction of ROS may result in cell and tissue injury and contribute to oxidative stress and chronic inflammation as the underlying pathophysiological mechanisms of a wide spectrum of pathological conditions related to neurodegeneration, atherosclerosis, metabolic diseases, carcinogenesis, or ageing [4,5,6,7,8].

The relationship between oxidative stress and microbiota dysbiosis has been a focus of increasing interest. The intestinal microbiota performs multiple functions related to signaling pathways and maintenance of homeostasis, interacting with nutrients and drug metabolism, performing intestinal barrier functions, protecting against pathogen colonization, and also working together with the immune system [9,10]. Excessive bioavailability of ROS may result from a disturbance of gut microbiota, contributing to an increase of oxidative stress. It has been shown that microbial-elicited ROS modulates innate immune signaling and mediates motility and increased cellular proliferation [11]. It has been hypothesized that at least partially-mediated ROS-dependent mechanisms are involved in potential beneficial effects of candidate probiotic bacteria as well as in many of the known effects of the normal microbiota on intestinal physiology [12]. Recent studies have shown fecal microbiota transplantation to be effective in the modulation of oxidative stress and reduced inflammation. A variety of mechanisms has been identified for the antioxidant action induced by probiotic bacteria in the gut. These include release of antioxidant molecules (e.g., glutathione) and secretion of antioxidant enzymes, direct ROS scavenging action, and their role as strong chelators of free copper or iron ions to prevent metal ion-catalyzed oxidation [13,14]. Probiotic exposure has also been associated with reduction of the activity of ROS-releasing enzyme systems such as NADPH oxidases and induction of cellular antioxidant signaling pathways such Nrf2-Keap1-ARE [15]. Altogether, it seems plausible that strategies able to impact the microbiome could potentially have an effect on oxidative stress.

On the other hand, intense physical exercise has been shown to be associated with different physiological changes, some of which include glucose and fatty acid oxidation, oxidative phosphorylation, and increased production of ROS and reactive oxygen nitrogen species (RONS) [16,17]. Additionally, gastrointestinal hypoxia and hypoperfusion during endurance exercise may increase intestinal permeability and oxidative stress in the gastrointestinal tract. Exercise-induced oxidative stress is affected by important factors, such as duration and intensity of exercise, training status, and nutritional intake. The effects of antioxidant intake (e.g., vitamin C, vitamin E, polyphenols, resveratrol, β-carotene, *N*-acetylcysteine) on exercise-induced oxidative stress have also been assessed in numerous experimental and human studies [18,19,20]. However, evidence of improvement of exercise performance or reduced muscle damage is inconsistent due to differences in the conditions of the exercise protocol and the administration of the antioxidant product (i.e., type, dose, timing, duration, etc.).

Based on the potential effects of probiotics as inducers of an antioxidant action and the increased production of ROS elicited by intense physical exercise, this study was conducted to test the hypothesis that supplementation with a probiotic product may be associated with beneficial effects in an oxidative stress model induced by high-intensity and duration physical exercise in male cyclists. Changes in gut bacterial microbiome were also examined.

## 2. Materials and Methods

### 2.1. Design

Between July 2018 and January 2019, a randomized, parallel-group, double-blind, placebo-controlled, and single-center trial was conducted at the Health Sciences Department of the Saint Anthony Catholic University (UCAM) in Murcia, Spain. The primary objective of this study was to evaluate the effect of the administration for 6 weeks of a daily regimen of a probiotic product, obtained from the mixture of three lyophilized probiotic strains, on an oxidative stress model based on the performance of physical exercise of high intensity and duration. The secondary objective was the evaluation of changes in bacterial microbiome from fecal samples. The study protocol was approved by the Ethics Committee of UCAM. Written informed consent was obtained from all participants. The study was registered in ClinicalTrials.gov (accessed on 18 February 2021) (NCT03798821).

### 2.2. Eligibility Criteria and Randomization

Caucasian healthy male volunteers aged 18–45 years who performed aerobic physical exercise between 2 and 4 times a week were eligible provided that they gave the written informed consent and none of the following exclusion criteria were present: history of chronic disease, particularly gastrointestinal disorders; abdominal surgery in last 3 months; asthma; chronic obstructive pulmonary disease (COPD); hypertension; sinus bradycardia; heart failure or cardiogenic shock; current smoking (>10 cigarettes/day); body mass index (BMI) > 30 kg/m^2^; alcohol or drug abuse; and poor tolerance or hypersensitivity to any component of the study product. The database of the Health Sciences Department of UCAM was used for the recruitment of participants.

Randomization (1:1) to supplementation with the probiotic product (probiotic group) or placebo (placebo group) was performed by an independent researcher using a random sequence of computer-generated numbers.

### 2.3. Intervention

Participants were given the probiotic product (300 mg capsules with 100 mg probiotic and maltodextrin and sucrose as carriers, 200 mg) or placebo (300 mg capsules with maltodextrin and sucrose) during 6 weeks. The probiotic product obtained from ADM-Biopolis (Valencia, Spain) was based on a mixture of three lyophilized probiotic strains: *Bifidobacterium longum* CECT 7347, *Lactobacillus casei* CECT 9104, and *Lactobacillus rhamnosus* CECT 8361 (in a ratio 1:4.5:4.5, 1 × 10^9^ total colony-forming units (cfu) per capsule). Participants were recommended to take one daily capsule, at breakfast, for 6 weeks. For all the strains a safety study including in vivo acute oral toxicity was previously evaluated, following the method described by Chenoll et al. [21] for *L. rhamnosus* CECT 8361 and *L. casei* CECT 9104 (data not shown).

### 2.4. Physical Exercise Oxidative Stress Model

The model was a high-intensity and long-lasting physical activity (90 min) on a bicycle roller. Participants underwent a preliminary test and two subsequent tests (test #1 after a 7-day washout period and test #2 at the end of the study at 6 weeks). The preliminary test was performed to calculate the intensity of tests #1 and #2 for each individual, using a bicycle roller with electromagnetic resistance (Technogym Spin Trainer) with an initial speed load of 12 km/h, with a 2 km/h load increase every minute, maintaining a constant slope of 2%. The cyclists employed free development. In order to calculate the intensity of tests #1 and #2, participants were monitored by ECG and gas analyzer (Jaeger Oxicom Pro^®^, CareFusion Respiratory Care, Germany) to determine maximal heart rate (MHR) and monitor heart rate above anaerobic threshold and during maximum oxygen uptake (VO_2_ max). Tests #1 and #2 lasted 90 min, and the maximum maintained load was equivalent to a heart rate corresponding to 75% of VO_2_ max calculated in the preliminary test. A constant slope of 2% was also used. The water consumption was ad libitum. After test #1, participants were given the assigned supplement (probiotic or placebo). Forty-eight hours before each test participants did not make any intense physical or psychological effort.

### 2.5. Study Procedures

The study included three visits, one at baseline during the time of the preliminary test, one at the time of test #1, and a final visit after test #2 at 6 weeks. At baseline, participants signed the informed consent, when eligibility criteria were checked, and the study product was given. Clinical evaluations included detailed medical history and measurement of anthropometric variables. Compliance with the intake of the probiotic product was assessed by counting the remaining capsules in the medication container. Adverse events were ascertained by directly asking participants how they were feeling after taking the product and from abnormal changes of laboratory results. During the study period, there were no dietary restrictions, but medications that may affect the microbiome (e.g., antioxidants, statins) were not allowed.

Peripheral blood samples (12 mL) after 12 h fasting were extracted at 30 min before and after each test, and 24 h urine samples were collected one day before and after the test. From the total urine volume, a 9 mL sample was frozen at −80 °C for further analysis.

Stool samples were collected during 24 h before test #1 and at 6 weeks during the 24 h before test #2, preserved with REAL stock buffer (Durviz S.L., Paterna, Valencia, Spain), and stored at −80 °C until analysis.

### 2.6. Study Variables

Body weight, BMI, and free fat mass were measured using bioelectrical impedance analysis (BIA) on a whole body BIA analyzer (Tanita BC-420MA, Tanita Corporation, Tokyo, Japan). Biochemical analyses included urinary isoprostanes (8-iso-PGF2α, ELISA kit, Oxford Biomedical Research, Rochester Hills, MI, USA), serum malondialdehyde (MDA) (MDA ELISA kit, Elabscience, Houston, TX, USA), and serum oxidized low-density lipoprotein (Ox-LDL) (Human OxLDL ELISA kit, Elabscience) as lipid-related oxidative stress biomarker; urinary 8-hydroxy-2′-deoxiguanosine (8-OHdG) (ELISA kit, Elabscience) as DNA-related oxidative stress biomarker, and serum protein carbonyl (Protein Carbonyl ELISA kit, Enzo Life Sciences, Lausanne, Switzerland) as protein-related oxidative stress biomarker; and serum glutathione peroxidase (GPx) (ELISA kit, Elabscience) and serum superoxide dismutase (SOD) (ELISA kit, Elabscience) as endogenous antioxidative enzymes. Safety analyses included complete blood count, liver function tests (bilirubin, alanine and aspartate aminotransferases, gamma-glutamyl transpeptidase), and renal function tests (blood urea nitrogen and serum creatinine levels).

For microbiome analysis, DNA was isolated with the aid of a QIAmp Power Fecal Pro DNA kit (Qiagen, Hilden, Germany), with bead beating and enzymatic lysis steps prior to extraction to avoid bias in DNA purification toward misrepresentation of Gram-positive bacteria. Massive genome sequencing of the hypervariable region V3–V4 of the bacterial 16s rRNA gene was conducted to evaluate the bacterial composition of the gut microbiome. Samples were amplified using key-tagged eubacterial primers [22] and sequenced with a MiSeq Illumina Platform, following the Illumina recommendations for library preparation and sequencing for metagenomic studies. The resulting sequences were split per patient, considering the barcode introduced during the PCR reaction. R1 and R2 reads were overlapped using PEAR program version 0.9.1, with an overlap of 50 nucleotides and a quality of overlap with a minimum of Q20, providing a single FASTQ file for each of the samples. Quality control of the sequences was performed by initial quality filtering (minimum threshold of Q20) using fastx tool kit version 0.013, followed by primer (16s rRNA primers) trimming and length selection (reads over 300 nts) with cutadapt version 1.4.126. These FASTQ files were then converted to FASTA files, and chimeras that could arise during the amplification and sequencing steps were removed by the UCHIME program, version 7.0.1001. Those clean FASTA files were BLAST against the National Center for Biotechnology Information (NCBI) 16s rRNA database using blastn version 2.2.29+. The resulting XML files were processed using a python script developed by ADM-Biopolis; (Valencia, Spain) to annotate each sequence at different phylogenetic levels.

### 2.7. Statistical Analysis

Analyses were performed in the per-protocol (PP) data set, which included all participants who completed the 6-week study period and underwent tests #1 and #2. The sample size was calculated for an expected mean difference between groups in serum levels of MDA of 1.34 nmol/mL with a standard deviation of 1.6 nmol/L according to data of Krotkiewsky et al. [23], so that for a significance level of 5% and statistical power of 80% assuming a drop-out rate of 10% since the primary analysis was performed in the PP data set, 20 evaluable participants for each treatment group were required. Categorical variables were expressed as frequencies and percentages, and continuous variables as mean and standard error (SE). Mean differences and 95% confidence intervals (CI) were calculated for changes between data at 6 weeks as compared with baseline. The chi-square (χ^2^) test or the Fisher’s exact probability test was used for the comparison of categorical variables between the probiotic and placebo groups. Quantitative variables were assessed using the analysis of variance (ANOVA) for repeated measures with three factors: time (baseline and final), test (test #1 and test #2) as within-subject factors and intervention (probiotic and placebo) as between-subject factor, with Bonferroni’s correction for pairwise comparisons.

In the case of microbiome analysis, alpha diversity was conducted using the vegan package, and statistical significance analyzed with the ANOVA test. The DESeq2 package from R (R Core Team, 2012) was used to generate a generalized linear model with fixed effects with negative binomial family, and the Wald test was used to compare operational taxonomic unit (OTU) counts between groups.

Statistical significance was set at *p* < 0.05. The SPSS software version 21.0 (IMB Corp., Armonk, NY, USA) was used for statistical analysis.

## 3. Results

### 3.1. Study Population

**Figure 1:**
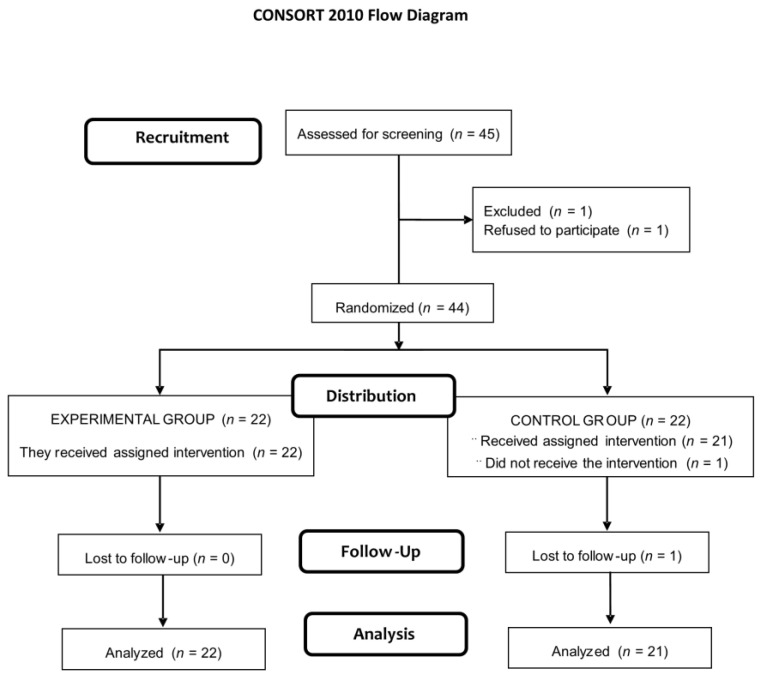
Flow chart of the study population.

Of a total of 45 eligible subjects, 1 declined to participate. The remaining 44 were randomized to the study groups (22 in each group), but 1 subject assigned to the placebo group did not receive the assigned intervention and was lost to follow-up. The final study sample included 22 subjects in the probiotic group (25.3 ± 7.2 years) and 21 (27.1 ± 8.4 years) in the placebo group (Figure 1). Baseline BMI was 23.6 (2.6) kg/m^2^) and VO_2_ max 51.1 (8.8) mL/kg/min. Significant differences after randomization were not observed.

### 3.2. Lipid, Protein, and DNA-Related Oxidative Stress Biomarkers and Antioxidative Enzymes

The oxidative stress model based on the performance of high intensity exercise and duration (test 1) produced statistically significant increases in biomarkers of oxidative stress and enzymes.

As shown in Table 1, urinary isoprostanes increased significantly in both groups after tests #1 and #2 as compared with baseline, but the difference between tests #1 and #2 (Δ mean difference) and between-group differences were not significant. Serum MDA showed a significant Δ mean difference of −207 ng/mL (95% CI −349.1 to 66.1) (*p* < 0.05) in the probiotic group only, with between-group differences also statistically significant (*p* < 0.05). Serum Ox-LDL showed a significant Δ mean difference of −122.5 pg/mL (95% CI −240 to −4.5) (*p* < 0.05) in the probiotic group only, but between-group differences almost reached statistical significance (*p* = 0.063). Urinary 8-OHdG increased significantly in both groups after tests #1 and #2, although the Δ mean difference (−10.9 pg/day, 95% CI −14.5 to −7.3; *p* < 0.001) was only significant in the probiotic group; moreover, between-group differences were also significant (*p* < 0.001). Serum protein carbonyl increased significantly after test #1 and test #2 in both groups, but neither Δ mean difference nor between-group differences were statistically significant. Serum GPx increased significantly in both groups after test #1 and in the placebo group only after test #2; however, neither Δ mean difference nor between-group differences were statistically significant. Serum SOD increased significantly in both groups after test #2, but again neither Δ mean difference nor between-group differences were statistically significant.

### 3.3. Microbiome Analysis

**Figure 2:**
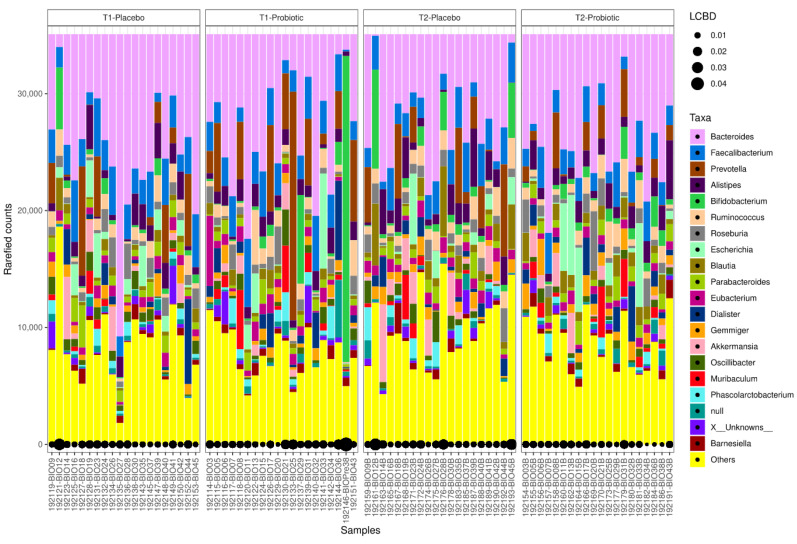
Local contributions to beta diversity (LCBD) analysis at family level (**right**) and genus level (**left**) from taxonomic identification of the samples sequenced (42 samples in the placebo group and 44 samples in the probiotic group; T1: before test #1, T2: at 6 weeks before test #2).

A total of 86 samples were included in the microbiome analysis (44 samples from participants in the probiotic group before test #1 (*n* = 22) and at 6 weeks before test #2 (*n* = 22), and 42 samples from participants in the placebo group before test #1 (n = 21) and at 6 weeks before test #2 (*n* = 21)). The local contributions to beta diversity (LCBD) at family and genus levels from taxonomic identification of the samples sequenced is shown in Figure 2.

**Figure 3:**
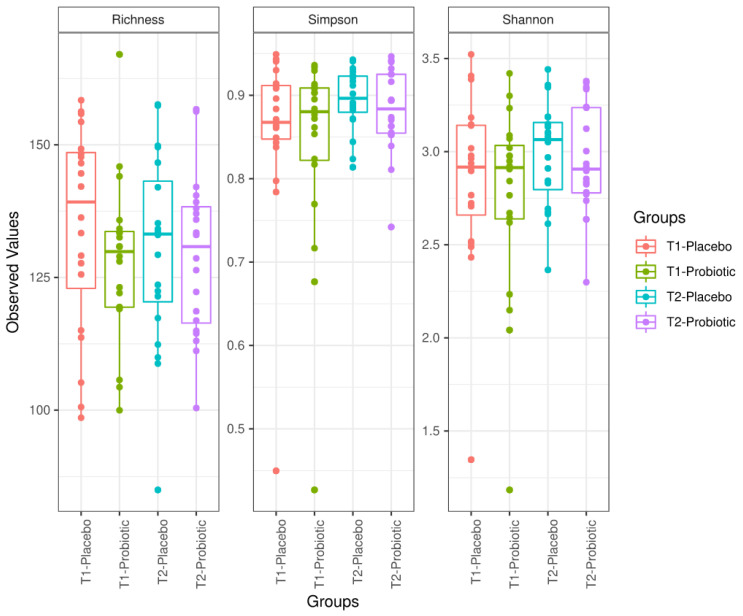
Richness, Simpson diversity index, and Shannon diversity index (from **left** to **right**) in the placebo and probiotic group at baseline (T1) and at 6 weeks (end of study) (T2).

Bacterial composition of samples was grouped, and both groups (placebo and probiotic) were compared at baseline and at 6 weeks at the end of the study. Richness, Simpson diversity index, and Shannon diversity index did not change after probiotic consumption (ANOVA test, *p* > 0.05 for all comparisons) (Figure 3).

**Figure 4:**
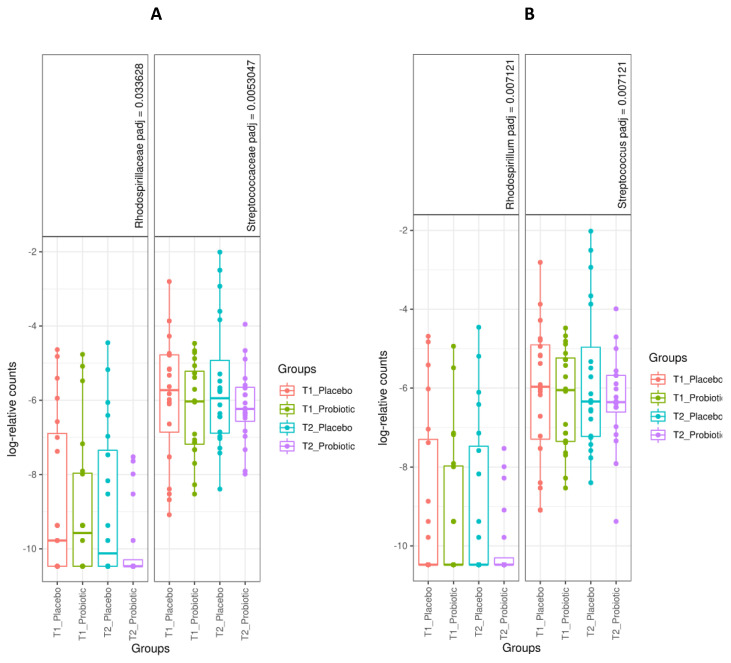
Differences between the placebo and probiotic groups at the end of the study (6 weeks) at the level of families (**A**) and genera (**B**).

Differences in bacterial population were measured with a Wald test using DESeq2 analysis. After 6 weeks of ingestion of the probiotic product or placebo (end of study), families Rhodospirillaceae (placebo vs. probiotic, log2 fold = 2.71, adjusted *p* value = 0.019) and Streptococcaceae (placebo vs. probiotic, log2 fold = 2.20, adjusted *p* value = 0.019) showed lower values in the probiotic group (Figure 4, left panel), considering a minimum threshold value of 10 counts (total average). There were statistically significant changes in seven genera, *Rhodospirillum* and *Streptococcus* being higher in the placebo group (Figure 4, right panel). However, within-group differences in the probiotic group showed an increase in specific genera, *Methanobrevibacter* (*M. smithii*), *Holdemanella* (*H. biformis*), and *Blautia* being the most remarkable, although *Lactobacillus* and *Lachnospira* decreased at the end of the study. Within-group differences in the placebo group revealed increases in *Bifidobacterium* and *Blautia*, among others, and decreases in *Shigella* and *Klebsiella* (in this case with low mean at baseline). Detailed data are shown in the Supplementary Materials, with Table S1 showing sequence distribution as well as sample metadata; Table S2 includes microbiome profiles at phylum, family, genus, and species levels, and Table S3 summarizes different populations at the genus level by Deseq2 analysis.

The probiotic product was well tolerated, and no adverse effects were observed. Additionally, laboratory tests at the end of the study did not show any abnormalities.

## 4. Discussion

In an oxidative stress model of high-intensity and duration physical exercise in male cyclists, daily intake of a probiotic product based on a mixture of *B. longum*, *L. casei* and *L. rhamnosus* for 6 weeks was associated with a significant reduction of lipid-related oxidative stress biomarkers, such as serum MDA, serum Ox-LDL, and DNA-related oxidative stress biomarker, such as urinary 8-OHdG. Several studies have shown that high-intensity and duration physical exercise results in oxidative stress, due to ROS being generated excessively by enhanced oxygen consumption, as well as in changes in muscle antioxidant enzyme activity [24,25,26,27]. Additionally, physical exercise models in endurance-trained competitive and non-competitive athletes have been used to assess the benefits of different supplements with antioxidant capacity [28,29,30,31,32].

Probiotic supplements are nutraceuticals with wide applications in different aspects of human health and have recently gained increasing interest for their potential effects as antioxidants due to anti-oxidative enzyme upregulation, stimulation of the production of a variety of bioactive peptides, and gut flora re-establishment [33]. However, there is limited evidence of the influence of probiotic supplementation on oxidative markers in athletes, and as far as we are aware there are only four studies examining antioxidant potential of probiotics in athletes. In a randomized double-blind, placebo-controlled study, 22 elite athletes received *Lactobacillus helveticus* (*n* = 10) or placebo (*n* = 12) for 3 months, and it a significant decrease of MDA and advanced oxidation protein products (AOPP) was found, without modifications in antioxidant enzyme SOD activity [34]. In a comparative study of two groups of 12 athletes each, probiotic supplementation with a combination of *Lactobacillus rhamnosus* IMC 501 and *Lactobacillus paracasei* IMC 502 administered for 4 weeks vs. no supplementation (controls) was associated with an increase in plasma antioxidant levels, thus neutralizing ROS [35]. A randomized, double-blinded, placebo controlled trial conducted in 23 trained men who received multi-species probiotics (*n* = 11) or placebo (*n* = 12) over 14 weeks, was designed to evaluate changes of markers of intestinal barrier, oxidation, and inflammation associated with the use of probiotic supplementation at rest and after intense exercise [36]. Participants performed a 90-min intense cycle ergometry at baseline and after 14 weeks. In this study, supplementation had no effect on protein carbonyl and MDA but decreased zonulin in feces as a marker, indicating enhanced gut permeability [36]. Finally, in a study of marathon runners, *Lactobacillus rhamnosus* GG (probiotic group) or placebo drink (placebo group) were given during the 3-month training period, 6-day preparation period, and marathon run, but probiotics did not show any effect on serum total antioxidant potential Ox-LDL [37]. However, studies requiring larger samples of athletes are needed to assess the beneficial role of probiotic supplementation on markers of oxidative stress damage.

On the other hand, other studies have examined the association between gut microbiota and oxidative stress in diseases in which oxidative stress plays a well-known pathogenetic role, such as type 2 diabetes mellitus. In a systematic review and meta-analysis of 13 randomized clinical trials involving 840 subjects, probiotics intake resulted in significant improvement in serum levels of total antioxidant status, MDA, and total glutathione (GSH), but there was a modest effect on serum glucose levels and glycated hemoglobin (HbA1c) [38]. Wang et al. [15] reported an in-depth review of the antioxidant mechanisms of probiotics, summarizing their involvement in decreasing radical generation and improving the antioxidant system based on modulation of the redox status of the host via their metal ion chelating ability, regulation of signaling pathways, antioxidant systems, ROS-producing enzymes, and gut microbiota.

A diversity of exogenous and endogenous stimuli are involved in complex molecular and cellular changes, including oxidative DNA damage and participation in cancer development [39], and different studies have explored the potential of probiotics (*L. casei* and *L. rhamnosus*) as cell-free supernatants to inhibit colon cancer cell invasion [40], the antiproliferative and apoptotic effects driven by *L. casei* ATCC 393 against experimental colon cancer [41], or *Lactobacilli* strains as modulators of *Fiaf* gene expression in human epithelial intestinal cells [42].

8-hydroxy-2′deoxiguanosine (8OHdG) is usually measured as an index of oxidative DNA damage [43,44] with oxidative modification of DNA that causes mutations during replication [45]. In recent years, there has been an increasing interest in the impact of exercise on epigenetic events; in particular, ROS-mediated methylation patterns are being investigated. The understanding of the mechanisms leading to ROS-associated epigenetic modifications may contribute to a better knowledge of carcinogenesis and its progression, together with discovering of implicated biomarkers [46,47].

An interesting aspect of the present study was the assessment of changes in microbiome besides improvement of biomarkers of oxidative damage induced by a model of high-intensity and duration physical exercise in response to supplementation with the probiotic product. The microbiota can be considered as a true endocrine organ, and the interactions between exercise and its adaptations, probiotics, and the microbiota itself could help athletes by producing beneficial metabolic, antioxidant, or anti-inflammatory effects that improve training. *Methanobrevibacter*, *Holdemanella*, and *Blautia* increased in participants consuming probiotics, whereas *Lactobacillus* and *Lachnospira* were within the taxa that decreased at the final point. *M. smithii* is a prominent microbe with methanogenic properties. In a humanized gnotobiotic mouse model of host–archaeal–bacterial mutualism, it was shown that *M. smithii* removed H_2_, which was related with more effective bacterial fermentation and subsequently more efficient short-chain fatty acids (SCFAs) production, increasing energy absorption [48,49]. *Holdemanella* is considered a butyrate producer. In a study of fecal microbiota collected from obese adults aimed to assess the effect of a pectin extracted from lemon and the probiotic strain *B. longum* BB-46, given in combination or alone, there was a positive correlation of *Holdemanella* with acetic and butyric acid, and a negative correlation with ammonium ions [50]. In an experimental high fat-induced oxidative stress, polyphenol supplementation affected different taxonomic levels of the gut microbiome by improving the proportion of *Blautia* (a butyrate producer) [51]. *Blautia* is one of the major taxonomic groups of the human gut microbiota (a genus in the *Lachnospiraceae* bacterial family, degrading complex polysaccharides to acetate, butyrate, and propionate (short chain fatty acids) that can be used by the host for energy and as a source of butyrate [52]. In a study of subjects who completed a 6-week endurance-based exercise intervention, there was an increase in butyrate concentrations induced by the exercise as a result of an increase in *Lachnospira* spp. [53]. This increase was independent of the BMI and decreased after return to sedentary activity. Surprisingly, *Lactobacillus* was found to be decreased at the end of the study, even being part of the probiotic. The reason for this finding is unknown. A point to be considered is how these strains could be able to resist the digestive system and arrive in sufficient amounts to detect enrichment of this genus. Conversely, both were detected by species-specific PCR in preliminary acute ingestion assays in feces (data not shown), although these results cannot be directly extrapolated to humans. Discussing a possible explanation for the functional effect of the formulation, even with a decrease in lactobacilli relative levels, is the potential capacity of extracellular metabolites of lactic acid bacteria to act as a prebiotic for key bacteria, influencing not only their growth and cell death, but also the expression of genes related to cell protection [54]. However, it seems that changes in microbiome do not directly correlate with the strains consumed, pointing that other mechanisms not necessary based on simple colonization might have a role on the results obtained.

The mechanisms by which the microbiome can impact upon oxidative stress and its effects are diverse. Among these, the production by the microbiota of toxic compounds can have a key impact on the health of the individual. Within this group, tryptophan catabolism by tryptophanase of certain bacterial groups produces indole, which is metabolized further to indoxyl-sulfate or indole-3 acetic acid. The latter toxins are secreted into the urine and are accumulated in the case of renal failure. These toxins decrease glutathione levels in renal tubular epithelial cells ren-dering them more vulnerable to oxidative stress [55]. Also, by activating ar-yl-hydrocarbon receptor (AhR) they can exert various deleterious effects [56,57].

Short-chain fatty acids, products of bacterial metabolism, have also been identified as an oxidative stress control mechanism. In a model of apoptosis in β-cells, butyrate and acetate attenuated the overproduction of ROS and NO and prevented cell apoptosis, and reduced viability and mitochondrial dysfunction [58]. Moreover, a bidirectional connection between mitochondrial genotype, ROS production, and gut microbiome has been recently established [59].

The present findings should be interpreted taking into account the limitations of the study, such as the small study population and the short duration of the intervention of only 6 weeks. Therefore, further studies with a larger sample size and duration of consumption of the probiotic product are warranted. It should be noted that in the present study, SOD and GPx were measured in serum samples, and significant differences between the study groups were not observed. However, it may be possible that significant differences could have been obtained by measurement of SOD and GPx in red blood cells.

## 5. Conclusions

Consumption of a probiotic product based on the three strains of *B. longum*, *L. casei*, and *L. rhamnosus* for 6 weeks in male amateur cyclists undergoing high-intensity and duration physical exercise was associated with a reduction of lipid-related oxidative stress biomarkers, without an increase in antioxidative enzymes. These findings suggest an antioxidant effect of the probiotic product on underlying interacting oxidative stress mechanisms and their modulation in healthy subjects.

## Figures and Tables

**Table 1:** Results of lipid, protein, and DNA-related oxidative stress biomarkers and antioxidative enzymes.

Variables	Test #1			Test #2 (6-Week Probiotic/Placebo Intake)			Test #1 vs. Test #2	Between-Group Difference *p* Value F Snedecor
	Baseline Mean (SE)	After Exercise Mean (SE)	Mean Difference (95% CI), *p* Value	Baseline Mean (SE)	After Exercise Mean (SE)	Mean Difference (95% CI), *p* Value	Δ Mean Difference (95% CI) *p* Value	
Urinary isoprostane, pg/day								
Placebo group	1.3 (0.5)	2.5 (0.7)	1.2 (0.5 to 1.9) *p* = 0.05	1.2 (0.5)	2.1 (0.7)	0.9 (0.3 to 1.5) *p* < 0.05	–0.3 (−0.8 to 0.2) *p* = 0.292	*p* = 0.213 F = 1.601
Probiotic group	2.1 (0.5)	3.3 (0.7)	1.3 (0.6 to 2.0) *p* < 0.05	2.2 (0.5)	3.6 (0.7)	1.4 (0.9 to 2.0) *p* < 0.001	0.1 (−0.3 to 0.7) *p* = 0.476	
Serum MDA, ng/mL								
Placebo group	347.4 (84.8)	491.1 (145.3)	143.7 (−25.8 to 313.2) *p* = 0.094	312.9 (64.3)	454.4 (113.3)	141.5 (−52.8 to 335.8) *p* = 0.149	−2.2 (−147 to 142.6) *p* = 0.975	*p* < 0.05 F = 4.195
Probiotic group	433.2 (82.9)	687.4 (142.0)	254 (88 to 419.8) *p* < 0.05	358 (62.9)	404.6 (110.7)	46.6 (−143 to 236.4) *p* = 0.623	–207.6 (−0.341 to −66.1) *p* < 0.05	
Serum Ox-LDL, pg/mL								
Placebo group	740.3 (82.9)	899.6 (64.1)	159.3 (81.9 to 236.7) *p* < 0.001	779.9 (64.2)	977.4 (78.4)	196.6 (83.0 to 310.2) *p* < 0.05	37.3 (−83.5 to 158.0) *p* = 0.536	*p* < 0.063 F = 3.653
Probiotic group	646.2 (60.1)	809.0 (62.6)	162.9 (87.2 to 238.5) *p* < 0.001	772.9 (67.6)	813.3 (77.1)	40.4 (−70.6 to 151.4) *p* = 0.467	−122.5 (−240 to −4.5) *p* < 0.05	
Urinary 8-OHdG, pg/day								
Placebo group	10.7 (0.2)	23.1 (3.8)	12.4 (8.3 to 16.6) *p* < 0.001	11.8 (2.4)	23.4 (3.3)	11.5 (8.1 to 15.0) *p* < 0.001	−0.9 (−4.6 to 2.8) *p* = 0.620	*p* < 0.001 F = 15.144
Probiotic group	13.3 (2.0)	29.0 (3.7)	15.7 (11.6 to 19.7) *p* < 0.001	13.6 (2.4)	18.4 (3.2)	4.8 (1.4 to 8.1) *p* < 0.001	−10.9 (−14.5 to −7.3) *p* < 0.01	
Serum protein carbonyl, pmol/mg protein								
Placebo group	124.0 (16.3)	160.0 (18.0)	36.0 (18.4 to 53.6) *p* < 0.001	112.4 (19.9)	162.0 (20.6)	49.6 (32.6 to 66.2) *p* < 0.001	13.6 (−4.4 to 31.6) *p* = 0.135	*p* = 0.434 F = 0.625
Probiotic group	166.8 (15.9)	204.2 (17.6)	37.4 (20.1 to 54.6) *p* < 0.001	162.9 (17.5)	204 (20.1)	41.1 (24.8 to 57.3) *p* < 0.001	3.7 (−13.9 to 21.3) *p* = 0.671	
Serum GPx, pg/mL								
Placebo group	526.9 (84.9)	788.0 (92.1)	261.1 (162.2 to 360.0) *p* < 0.001	633.8 (80.3)	1111.7 (214.5)	477.8 (112.5 to 843.2) *p* < 0.05	216.7 (−156.4 to 598.9) *p* = 0.248	*p* = 0.253 F = 1.598
Probiotic group	473.4 (83.0)	594.8 (90.0)	121.4 (24.7 to 218.0) *p* < 0.05	598.5 (78.4)	610.0 (209.6)	11.6 (−345.4 to 368.5) *p* = 0.948	−109.9 (−474.4 to 254.7) *p* = 0.546	
Serum SOD, ng/mL								
Placebo group	24.1 (2.6)	34.5 (3.3)	10.5 (5.9 to 15.1) *p* < 0.001	22.1 (2.2)	29.9 (2.7)	7.8 (3.7 to 11.9) *p* < 0.001	−2.9 (−8.6 to 3.2) *p* = 0.358	*p* = 0.267 F = 1.274
Probiotic group	29.2 (2.6)	33.1 (3.3)	3.9 (−0.7 to 8.5) *p* = 0.094	24.9 (2.2)	30.7 (2.7)	5.8 (1.7 to 10) *p* < 0.05	2 (−4 to 7.8) *p* = 0.511	

SE: standard error; CI: confidence interval; MDA: malondialdehyde; Ox-LDL: oxidized low-density lipoprotein; GPx: glutathione peroxidase; SOD: superoxide dismutase; F: F-Snedecor.

## Data Availability

No new data were created or analyzed in this study. Data sharing is not applicable to this article.

## Supplementary Materials

The following are available online at https://www.mdpi.com/2076-3921/10/2/323/s1, Table S1: Sequences_distribution; Table S2: Microbiome_profile; Table S3: DESeq2_differential-populationGenus.
